# MetAssimulo:Simulation of Realistic NMR Metabolic Profiles

**DOI:** 10.1186/1471-2105-11-496

**Published:** 2010-10-06

**Authors:** Harriet J Muncey, Rebecca Jones, Maria De Iorio, Timothy MD Ebbels

**Affiliations:** 1 Department of Epidemiology and Biostatistics, School of Public Health, Imperial College London, UK; 2 Biomolecular Medicine, Department of Surgery and Cancer, Imperial College London, UK

## Abstract

**Background:**

Probing the complex fusion of genetic and environmental interactions, metabolic profiling (or metabolomics/metabonomics), the study of small molecules involved in metabolic reactions, is a rapidly expanding 'omics' field. A major technique for capturing metabolite data is ^1^H-NMR spectroscopy and this yields highly complex profiles that require sophisticated statistical analysis methods. However, experimental data is difficult to control and expensive to obtain. Thus data simulation is a productive route to aid algorithm development.

**Results:**

MetAssimulo is a MATLAB-based package that has been developed to simulate ^1^H-NMR spectra of complex mixtures such as metabolic profiles. Drawing data from a metabolite standard spectral database in conjunction with concentration information input by the user or constructed automatically from the Human Metabolome Database, MetAssimulo is able to create realistic metabolic profiles containing large numbers of metabolites with a range of user-defined properties. Current features include the simulation of two groups ('case' and 'control') specified by means and standard deviations of concentrations for each metabolite. The software enables addition of spectral noise with a realistic autocorrelation structure at user controllable levels. A crucial feature of the algorithm is its ability to simulate both intra- and inter-metabolite correlations, the analysis of which is fundamental to many techniques in the field. Further, MetAssimulo is able to simulate shifts in NMR peak positions that result from matrix effects such as pH differences which are often observed in metabolic NMR spectra and pose serious challenges for statistical algorithms.

**Conclusions:**

No other software is currently able to simulate NMR metabolic profiles with such complexity and flexibility. This paper describes the algorithm behind MetAssimulo and demonstrates how it can be used to simulate realistic NMR metabolic profiles with which to develop and test new data analysis techniques. MetAssimulo is freely available for academic use at http://cisbic.bioinformatics.ic.ac.uk/metassimulo/.

## Background

In the postgenomic era there has been a massive growth in 'omics' techniques investigating different levels of biological organisation. Metabolic profiling (or metabonomics/metabolomics) is a key area of systems biology research focussing on high-throughput identification and quantification of metabolites, small molecules (≤ 1500 Da) involved in metabolism [1]. When trying to relate genes to the overall function of a system, the metabolome (the complete set of metabolites) more closely reflects the activities of the organism at a functional level than, for example, the transcriptome [2]. Metabolic fluxes are not only regulated by gene expression, but also by additional factors, which include the abundance of metabolites as substrates and products [3]. Therefore metabolic profiling adds another dimension to our understanding of biological systems.

A commonly used form of analysis in metabolic profiling is ^1^H Nuclear Magnetic Resonance (NMR) spectroscopy of biofluids. Metabolites in biofluids are in dynamic equilibrium with those in cells and tissues so their metabolic profile reflects changes in the state of an organism due to disease or environmental effects. ^1^H-NMR spectroscopy gives a global metabolic profile as it has the potential to detect nearly all proton-containing metabolites. Despite relatively poor sensitivity in comparison with analytical methods such as mass spectrometry, NMR spectroscopy requires minimal sample preparation and is able to measure concentrations as low as 100 *μ*M [4] and even lower with recent techniques such as cryoprobe technology. NMR allows metabolites to be detected simultaneously without preselection. The NMR spectrum for each metabolite is comprised of a characteristic pattern of peaks or resonances, derived from three main factors:

1. The chemical shift (*δ*) of each resonance is dependent upon the local magnetic field experienced by each nucleus. This local field is dependent on the degree to which molecular orbitals shield the influence of the external spectrometer field. Thus the chemical shift can reflect the chemical structure of the metabolite. The position of each peak is measured relative to that of an internal standard in a scale of parts per million (ppm) [5]. A commonly used internal standard is 3-(Trimethylsilyl)-Propionic acid-D4, sodium salt (TSP).

2. Spin-spin coupling causes NMR resonances to split into multiplet patterns due to magnetic interactions between nearby nuclei.

3.Integrated peak area is proportional to the number of observed ^1^H nuclei (assuming there are no differential relaxation effects) and allows quantification of the metabolite concentrations.

The NMR spectrum of a complex mixture can be well approximmated by a linear combination of the spectra of pure compounds, potentially thousands of metabolites. Biofluid spectra can be treated as *K *dimensional objects, in which each dimension represents the concentration of a single metabolite [6]. This super-posed structure is exploited in our simulation method, detailed in the 'Implementation' section.

Metabolic NMR spectra are highly complex and the field benefits greatly from the application of machine learning and statistical tools to extract information. Pattern recognition analyses such as Principal Components Analysis (PCA) have long been combined with NMR to investigate normal and pathological metabolic states [7]. Data processing methods are being developed to extract metabolite information and concentrations from raw spectra, allowing automation of spectral processing. Development of advanced mathematical, statistical and computational methods are also essential for characterisation of the metabolic state, delineation of metabolic changes over time and the efficient identification of potential biomarkers. There are a wide variety of diseases where key changes in metabolites have been deduced e.g. cancer, diabetes, hypertension etc. [8-10]. However, as algorithms and methods are developed, they need to be refined and validated to ensure results will be biologically meaningful. It is hard to effect this without using test datasets where the true answers are known; this can be accomplished using simulation techniques. An alternative approach is to design artificial mixtures of metabolites which are prepared and analysed in identical fashion to real samples. However this is expensive in terms of man power and instrument time, and offers few advantages over in silico simulation when assessment of analytical procedures is not required. The purpose of MetAssimulo is to simulate datasets of realistic NMR spectra with *known *parameters in order to test data analysis techniques, hypotheses and experimental designs. Few methods for generating simulated NMR datasets have appeared in the literature to date [11,12]. Most model a limited number of metabolites, make no attempt to reproduce realistic levels of metabolites, and do not allow for between-metabolite or 'inter-metabolite' correlations ([12] excepted) and do not always model peak positional shifts. It is common to fit Lorentzian peak shapes in an attempt to characterize spectral peaks, e.g [11]. However, this ignores the fact that peak shapes in real NMR profiles are variable and can be far from ideal. Here we outline a novel approach making use of individual standard metabolite data extracted from the Human Metabolome Database (HMDB) [13] and a local NMR standard spectra database (NSSD). Many metabolic profiling labs host their own NSSD appropriate to the biological systems and sample types they work with and thus the simulations can be tailored to virtually any sample type or organism as required. In this work human urine is used as the example biofluid as it is one of the most widely used in the field and, in healthy subjects, has no protein or lipid content, both of which make the simulation more complex. MetAssimulo is written in MATLAB with a graphical interface allowing the user to alter processing parameters and add new standard spectra as needed. The software is freely available along with an example NSSD of 48 metabolites commonly found in normal human urine. We stress that this list of metabolites and their concentration means and standard deviations does not constitute a *definitive *description of human urine; such a goal is beyond the scope of this paper. It is provided for the sole purpose of demonstrating the capabilities of the software.

## Implementation

**Figure 1:**
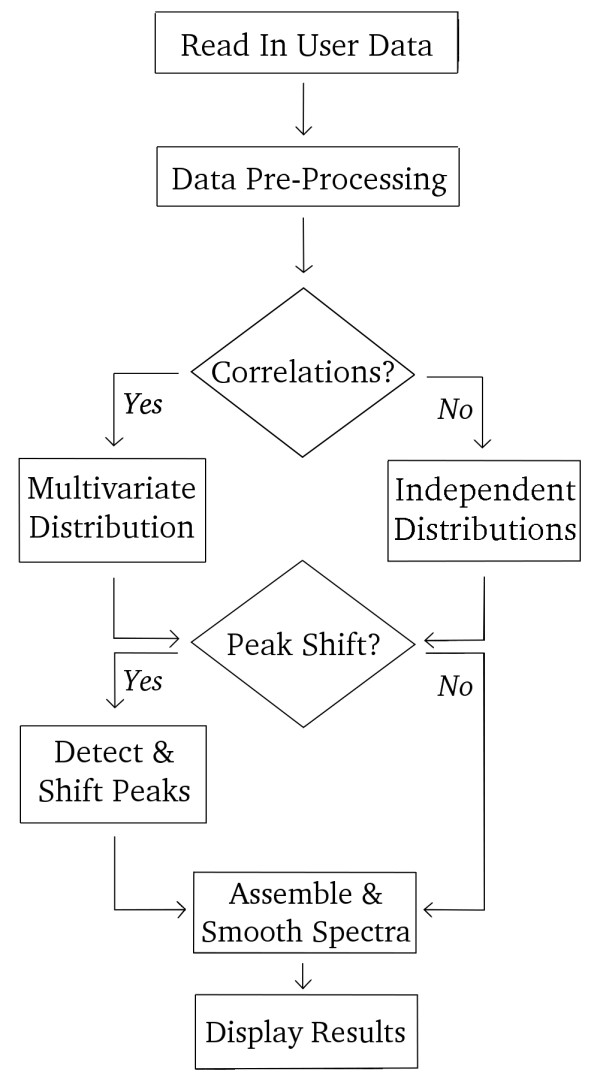
**MetAssimulo Structure**.

MetAssimulo performs various functions accessed though the Graphical User Interface (GUI): pre-processing the pure spectra, simulating metabolite concentrations, incorporating peak shifts and creating the final mixture spectrum (Fig. 1). By default it produces two groups of spectra based on different metabolite mixtures; these could represent controls (normal) and cases (diseased) subjects.

Each metabolite has a characteristic pattern of peaks on a linear scale, the chemical shift, given by *δ *in ppm. The signal intensity, *y*(*δ*), in a spectrum of metabolites *k *(*k *= 1, .., *K *where *K *is the total number of metabolites in the mixture) at a given *δ *increases proportionally to the concentration of each metabolite, *c_k_*, present in the sample and their number of observed protons, *p_k_*. The different metabolite spectra are summed together to produce the overall mixture spectrum. Normally distributed additive noise $\epsilon \left(\delta \right)∼N\left(0,\sigma_{n}^{2}\right)$ (see 'Calculate noise standard deviation' section for estimate of $\sigma_{n}^{2}$) is then added to the mixture spectrum (see Eqn.1), *y*(*δ*), which is then smoothed to simulate the conventional preprocessing technique of exponential apodization prior to Fourier Transform [14].

(1) $$y\left(\delta \right)=\sum_{k=1}^{K}\left(y_{k}\left(\delta \right)c_{k}p_{k}\right)+\epsilon \left(\delta \right)$$

Each individual metabolite spectrum is sampled at a series of *n *uniformly spaced data points. The overall spectrum is made up of pairs of data points, (*x*, *y*) = (*x_i_*,*y_i_*)_*i *= 1,..., *n*_, where *y_i _*= *y*(*x_i_*); *n *is set by the user, and *x_i _*defines a point on the ppm grid.

In real NMR spectra, the signal intensity is affected by the extent to which the observed nuclei are allowed to relax before each observation. In MetAssimulo we do not currently attempt to simulate the effects of differential inter-molecular relaxation. However, intra-molecular relaxation effects are accounted for by the fact that experimentally obtained pure compound spectra are used to form the mixture spectra.

### Setting Parameters

Parameters can be altered either in the MetAssimulo GUI or within the parameter file 'parameters.txt'. Default values for parameters are given in the Supplementary Material. The interface provides the user with several different processing options. For example the second group ('cases') may be specified as fold change ratios of the concentrations of the first group and the user can specify whether to produce output with or without peak shifts or both. The user also chooses whether to include inter-metabolite correlation (pairwise correlations between metabolites) or not; either as a textfile whose entries can be altered using the interface or constructed from scratch in the Correlation GUI.

#### Input Files

The Human Metabolome Database (HMDB) [13] contains information about more than 2180 metabolites found in humans and includes literature data relating to normal and abnormal concentrations in biofluids. Metabocards is the flat file download of the entire database, available at http://www.hmdb.ca. Also required is the HMDB set of NMR Peak Lists (containing locations of individual peaks for metabolites) which is available in a downloadable zip-file. In constructing the template of normal human urine concentrations various problems of incompleteness and/or ambiguity were encountered. For example, in many HMDB entries the metabolite concentration mean and standard deviation is unavailable, or simply a range is given. In these cases the standard deviation was estimated by dividing the mean (or 'half-range') by 1.95. There are instances where a metabolite is identified as present in urine, but a normal concentration value is not available. We have attempted to rectify as many of these discrepancies as possible in the provided concentration file by cross-checking with other sources, i.e. literature articles, but do not claim the result represents a complete description of human urine; it does, however serve to demonstrate the software.

The quality of MetAssimulo simulations is also dependent on the quality and coverage of the NSSD used, as well as the peak shift settings affecting multiplet detection. By distributing an NSSD it is not our intention to provide a comprehensive NMR standard database but merely an initial set of common metabolite spectra with which users can begin to make their own simulations. Many users will wish to add their own locally acquired standard spectra for metabolites specific to their areas of interest and we have provided functionality to do this.

There are a number of input files that are required for MetAssimulo.

Concentration files* are needed for both groups of metabolites, these detail the mean and standard deviation of the concentration for each metabolite.

**An NMR Standard Spectral Database (NSSD) **comprising standard 1D ^1^H-NMR spectra for metabolites is essential. MetAssimulo is designed to work with any metabolite database set out in the Bruker file format. Standard spectra of 48 of the most abundant metabolites in normal human urine is distributed with MetAssimulo.

**Experiment file **identifying the experiments to use in the metabolite database, as one metabolite may have many spectra, taken at different pH for example.

**Proton file **listing the number of protons, *p_k _*observed for each metabolite, *k*.

Multiplet data files* specifying the position of each peak in a multiplet for each metabolite in order to incorporate simulated peak shifts. Known pKa values and acid/base limits can also be included.

**Inter-metabolite correlations **can be input via a text file or the GUI.

Synonym files* that allow MetAssimulo to match metabolites in the HMDB data to those in the NSSD.

**Parameter file **containing the default values or simulation parameters (alterable in the GUI).

*Can be generated automatically using 'Format HMDB Data' within MetAssimulo.

Examples of all input files in the appropriate format are included with the MetAssimulo distribution. Much of the required input data can be generated using the in-built function 'Format HMDB Data' (accessed via the GUI) which should be run as an initial 'setup'. It produces the files necessary for conversion between the local database and HMDB synonyms, data required for peak shift simulation and a raw template of concentration data for 'normal' urine. The normal urine concentration file provided with the distribution has been optimized to provide realistic values and correct a number of errors found in the current version of the HMDB whilst reducing the number of metabolites used in order to decrease processing time.

### Pre-processing

Initially, a set of metabolite concentrations is simulated for the case and control groups, based on the mean and standard deviations in the concentration file. Next, the required spectra from the NSSD must be loaded. Even ^1^H-NMR spectra of standard pure compounds contain a number of complexities, such as chemical and electronic noise, phase and baseline errors, contaminants and water suppression residuals. Thus it is ususally necessary to preprocess these spectra into a form suitable for combining into the final metabolic profiles.

#### Simulating Concentrations

Concentrations, *c_k_*, for each metabolite, *k *= 1, .., *K*, are simulated for the number of replicates specified by the user. Individual metabolite concentrations are generated from a truncated normal distribution, Eqn.2, using the inverse cdf method since negative concentrations are unphysical [15]. Here *μ *is the mean concentration and *σ *is the standard deviation input by the user.

(2) $$c_{k}∼N\left(\mu ,\sigma^{2}\right)I\left(c_{k}>0\right)$$

Significant inter-metabolite correlations, here assumed to be linear pairwise correlations between metabolites, are often found within the field of metabolic profiling so were considered an important feature to incorporate into the simulation. Where inter-metabolite correlations are required, the concentrations are simulated by sampling from the appropriate multivariate normal distribution. Rejection sampling is utilised to ensure non-negative concentrations. Using the method detailed in [16] the nearest positive semidefinite correlation matrix is calculated given user-specified pairwise correlations. The covariance matrix is constructed using the metabolite standard deviations and specified correlations, and the diagonal entries are increased sufficiently to ensure positive-definiteness. Any necessary alterations to the correlation and covariance matrices are output for inspection.

#### Read in spectrum

After the concentrations have been simulated the standard spectra of the metabolites are read in. Each spectrum consists of chemical shift in ppm, *x *and intensity, *y*. Spectra are then linearly interpolated onto a ppm grid of user-specified resolution.

#### Exclusion regions

Exclusion regions, corresponding to the location of the internal standard peak (default < 0.2 ppm [17]) and the residual water peak (default 4.5 ppm - 6.0 ppm [17]), are set to zero. In urine, the urea signal (between 5.4 ppm and 6.0 ppm [17]), the most abundant proton-containing metabolite [6], can be problematic particularly when water-suppression methods are used. Water-suppression is usually imperfect and the resulting residual peaks (near to the urea signal) are not dealt with easily by baseline correction algorithms [18]. Often, the urea and water peaks are combined into one exclusion region lying between 4.5 ppm and 6 ppm (default exclusion region, but can be adjusted by the user). Excluding these areas of the spectrum helps reduce sensitivity to artifacts.

#### Baseline Correction

It is easier to distinguish peaks in a spectrum when the baseline is featureless [14], however, spectra can have distorted baselines due to imperfections in the detection process [17]. Curved baselines can be a major source of error and so a correction is carried out on the raw spectrum using a moving average [19]. This method involves splitting the data into windows of size *ω *(default is 0.3125 ppm), defined by the user, then using the median within the window to estimate the baseline. In order to alter the baseline without losing metabolite peaks, a threshold is set by dividing the maximum height by a user specified parameter (default is 10). All the intensities found below this threshold are corrected by subtracting the estimated baseline.

#### Removal of Negative Artifacts

Negative artifacts, produced by baseline correction or simply inherent in the original spectrum must be removed since their presence could interfere with peaks of interest in the mixture spectrum. This is remedied by using an estimate of the noise standard deviation, *σ_med_*, to calculate a limit value, *l*, using Eqn.3. *σ_med _*is estimated by splitting the spectrum into a number of bins (given by the user, default 32) and calculating their standard deviations. The median of these standard deviations is used as the estimate of *σ_med_*.

(3) $$l=M-3\sigma_{med}$$

*M *is the median of the intensities, *y_i_*. All intensities appearing below this limit,*l*, are set equal to it.

#### Kernel Smoothing

Noise from each standard metabolite spectrum will remain in the final mixture spectrum, reducing the overall signal to noise ratio. Kernel smoothing is used to reduce the noise in each individual metabolite spectrum. This process estimates the smooth function underlying the noisy data using a weighted mean of surrounding data points with weights defined according to the choice of kernel. Whilst the default kernel type is 'Normal', the user may also choose from a number of options and also alter the bandwidth (given as number of data points), controlling the degree of smoothing required. Since smoothing the whole spectrum woiuld increase the peak widths, only intensities below a user-defined threshold (a percentage of the maximum intensity, default 0.8) are subject to kernel smoothing.

#### Peak Shift

If the user has chosen to simulate peak shifts (this is the default setting), these are then calculated for each multiplet in each metabolite spectrum. First, a peak detection process is used to identify peaks suitable for shifting. Peaks detected are cross-referenced with the HMDB multiplet data to determine those belonging to a multiplet that must be shifted together. Whether or not a peak is shifted depends on the user defined thresholds for peak detection, and also its size relative to the noise. In real samples, peak positional variation can derive from various matrix effects primarily pH differences but also due to variation in the concentration of other ionic species in the mixture. In MetAssimulo, we take account of pH variation only; this is sufficient to produce very realistic shift patterns and avoids the need for many extra parameters in the model. If pKa values and acid and base limits are not available, values are drawn from normal distributions with user-specified mean and standard deviation. If the user requires the same pH for all replicates of a mixture, the pH value is set as the user input. Otherwise, the pH values are sampled from a normal distribution with mean and standard deviation defined by the user. This information is then combined using the Henderson-Hasselbalch Equation [5] (Eqn.4) to calculate the peak shift (in ppm) and the peaks are shifted accordingly.

(4) $$\eta_{ij}=\frac{e^{pH-pKa_{j}}(a_{ij}-\delta_{ij})-\delta_{ij}+b_{ij}}{e^{pH-pKa_{j}}+1}$$

where *δ_ij _*is the un-shifted position of peak *i *of metabolite *j *in ppm (known),

*η_ij _*is the amount the peak is shifted in ppm (generated by Eq.4),

*pH *is the pH of sample (simulated or input),

*pKa_j _*is the pKa of metabolite *j *(simulated or input) assumed here to be the same for all peaks of a given metabolite,

*a_ij _*is the position of peak *i *of metabolite *j *in the acid limit (ppm) (simulated or input),

*b_ij _*is the position of peak *i *of metabolite *j *in the basic limit (ppm) (simulated or input).

After this process, the spectrum is then smoothed again in order to suppress any unwanted artifacts created by the peak shift.

### Simulating Mixture Spectra

To make sure that all the metabolite spectra are on a comparable scale the spectra are normalised to unit integrated intensity, using Eqn.5.

(5) $$y_{i}=\frac{{\tilde{y}}_{i}}{\sum_{i=1}^{n}{\tilde{y}}_{i}}$$

where ${\tilde{y}}_{i}$ is the intensity in the preprocessed, unnormalised standard spectrum.

#### Calculate noise standard deviation

The final mixture spectrum is constructed using Eqn.1. The standard deviation *σ*_n _of noise to be added is calculated by dividing the maximum peak intensity by the signal to noise ratio required by the user, *SNR*, as in Eqn.6.

(6) $$\sigma_{n}=\frac{max\left(y\left(\delta \right)\right)}{SNR}$$

Even after preprocessing, the signal to noise levels of the individual metabolite spectra may vary, so therefore the final signal to noise ratio cannot be controlled perfectly. However, adding noise in this way allows the simulation of mixture spectra with a wide variety of signal to noise ratios. After adding the random noise, *ε*(*δ*) ~ *N *(0, *σ_n_*), kernel smoothing is used on the composite spectrum to reproduce the effect of apodization on real spectra [14].

## Results and Discussion

In this section some example outputs from MetAssimulo will be shown. Simulations of normal urine were run using the optimized template with parameters set to the default values and using the NSSD consisting of 48 spectra recorded at 600 MHz ^1^H observation frequency.

### Single Spectrum

**Figure 2:**
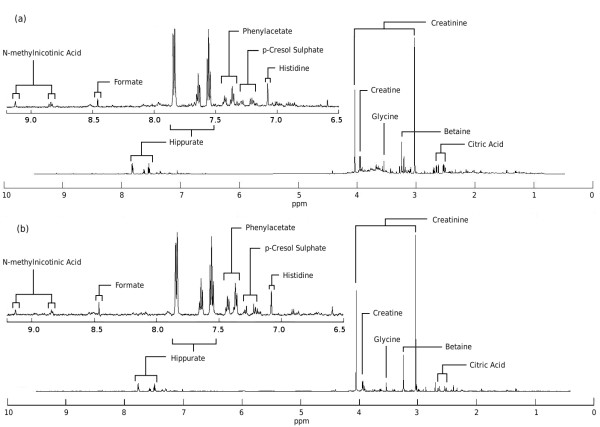
**(a) Real normal urine sample, (b) Mean simulated normal urine sample**.

To test whether MetAssimulo's output spectra (Fig. 2(a)) seem realistic they are compared to a real normal human urine spectrum with the same exclusion regions (Fig. 2(b)). It should be noted that differences between real and simulated spectra will result not only from the simulation process, but also from incomplete knowledge of the exact molecular species giving rise to NMR signals and uncertainties in their levels. However, despite these difficulties, the simulated and real spectra show many similarities including the dominance of high abundance metabolites such as creatinine, glycine, and citric acid. The insets show how such realistic simulation extends to low intensity signals of the aromatic region such as hippurate, histidine, formate and N-methylnicotinic acid.

### Simulation of Case & Control Groups

**Figure 3:**
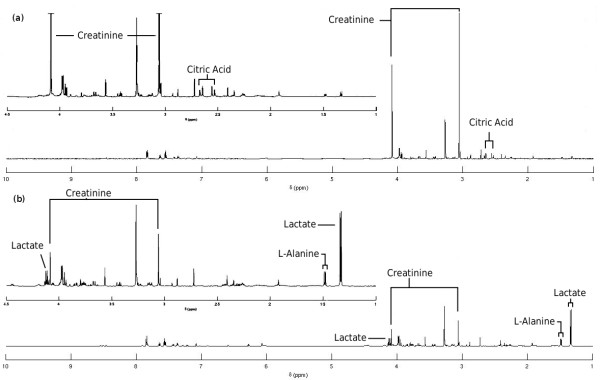
**(a) Mean simulated normal urine spectrum, (b) Mean simulated spectrum of urine in paraquat poisoning**.

**Figure 4:**
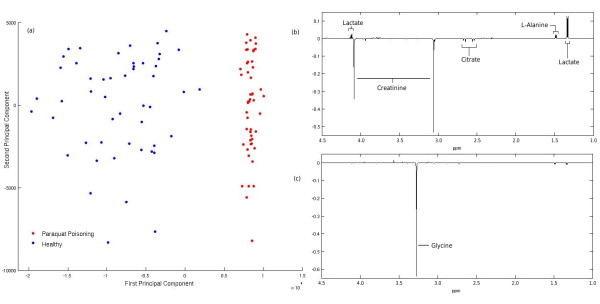
**(a) Scores plot of the first two principal components for the simulated data, (b) Loadings on PC1, (c) Loadings on PC2**.

We now show how MetAssimulo can produce two groups of spectra with different metabolite compositions. We simulated spectra for both normal urine and a diseased state. Paraquat poisoning [20] was chosen as the diseased state, from several available in the HMDB, because it shows a diverse array of metabolic disregulation in comparison with normal urine. The concentrations of 4 metabolites are altered: citric acid and creatinine are decreased, whilst alanine and lactic acid are increased. Simulations were run for 50 replicates of normal and diseased without peak shifts, the mean of which are shown in Fig. 3. These spectra clearly show the expected decrease in citric acid and creatinine concentrations for Paraquat poisoning, whilst alanine and lactate concentrations are increased. The PCA scores plot in Fig. 4(a) clearly demonstrates separation in the first principal component. The largest loadings on PC1, Fig. 4(b), correspond to the metabolites that were altered, accurately describing the difference between the two groups. The largest loading on PC2 corresponds to glycine, the metabolite with the highest with-group variance. This data could be used in disease diagnostics to help train machine learning methods in recognising disease status.

### Peak Shifts

**Figure 5:**
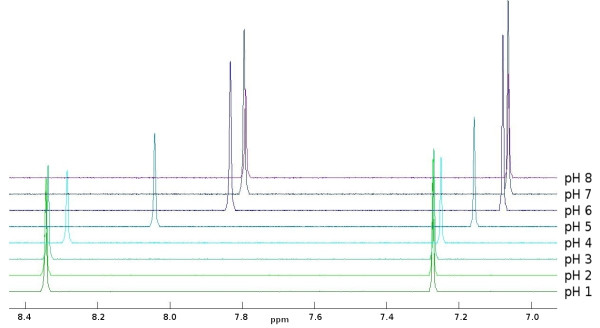
**Simulated peak shift for the two aromatic singlets of histidine**.

We demonstrate the peak shift using histidine, a metabolite particularly prone to this kind of positional variation. Acid and base limits were estimated by inspecting spectra taken at varying pH values. Fig. 5 clearly shows a shift in ppm values for this peak consistent with the non-linear mechanism described by the Henderson-Hasselbach Eqn.4.

### Inter-Metabolite Correlations

To demonstrate the incorporation of inter-metabolite correlations, we specify the pairwise Pearson correlations of three metabolites: citrate, creatinine and 2-oxoglutarate. The following correlation matrix was used.

$$\left(\begin{array}{cccc} citrate & 1 & & \\ creatinine & -0.7 & 1 & \\ 2-oxoglutarate & 0.8 & -0.4 & 1 \end{array}\right)$$

**Figure 6:**
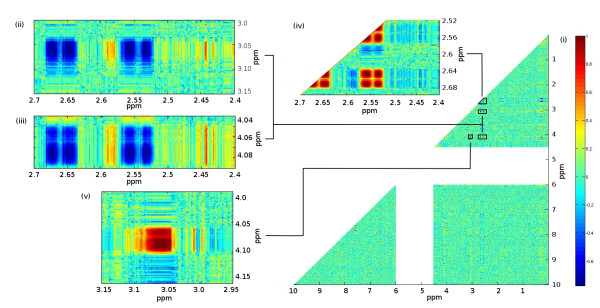
**Inter and intra-metabolite correlations: (i) Complete correlation matrix and insets (ii)-(v) showing strong negative inter-metabolite correlation between citrate and creatinine and positive between 2-oxoglutarate and creatinine (ii), (iii) and strong positive intra-metabolite correlations for creatinine (iv) and citrate (v)**. Colour scale indicates the level of Pearson correlation.

**Figure 7:**
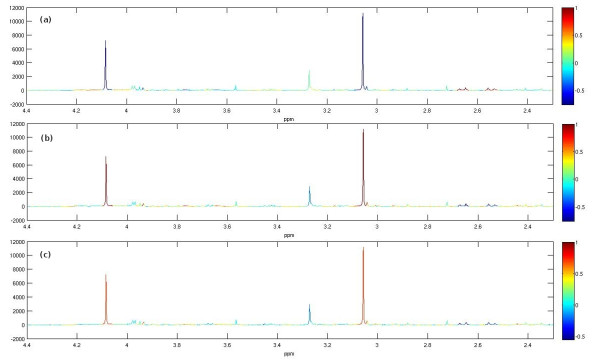
**Pairwise correlation coefficients mapped as a colour code onto the mean spectrum**. Correlations to (a) citrate 2.65 ppm, (b) creatinine 4.08 ppm, (c) 2-oxoglutarate 2.44 ppm.

This resulted in a positive definite covariance matrix, so no adjustments were required. Fig. 6(i) visualizes the correlation matrix between all spectral intensities. Most correlations are close to zero as expected. The regions enlarged in (ii)-(v) illustrate the the correlations that were expected. The correlations can also be viewed in Fig. 7 when the mean spectrum is coloured according to the correlation coefficient with respect to a specified chemical shift corresponding to a particular metabolite peak position ((a) citrate-2.65 ppm, (b) creatinine-4.08 ppm, (c) 2-oxoglutarate-2.44 ppm). Note that these analyses are similar to the commonly used STOCSY [21] technique which is used to analyse both inter- and intra-metabolite correlations; our simulations could be used to develop and test such methods.

## Conclusions

There are currently simulation programs in different areas of post-genomic science, such as SNP simulators that are being used in whole genome association studies [22,23]. MetAssimulo is a valuable addition to these tools, enabling the simulation of realistic ^1^H NMR spectra of complex biological mixtures including group-wise variation, intermetabolite correlations and peak positional variation. However, there are areas which could be enhanced. Any simulator of this kind is limited by the sources of data available. The HMDB only contains information about metabolite concentrations in humans, therefore further user input or other metabolite databases may be needed to address other organisms. Human urine is the default setting for MetAssimulo, but given the numerous alterable parameters, it is easy to simulate profiles for other species and biofluids.

## Availability and requirements

Project name: MetAssimulo

Project home page: http://cisbic.bioinformatics.ic.ac.uk/metassimulo/

Operating system(s): Platform independent

Programming language: MATLAB

Other requirements: MATLAB

## List of Abbreviations used

GUI: Graphical User Interface; HMDB: Human Metabolome Database; NMR: Nuclear Magnetic Spectroscopy; NSSD: NMR Standard Spectra Database.

## Authors' contributions

TE and MDI concieved the project and supervised the work and reviewed the manuscript. HM and RJ wrote and tested the code. HM wrote the manuscript. All authors reviewed and approved the maniscript.
